# GADD45a Regulates Olaquindox-Induced DNA Damage and S-Phase Arrest in Human Hepatoma G2 Cells via JNK/p38 Pathways

**DOI:** 10.3390/molecules22010124

**Published:** 2017-01-13

**Authors:** Daowen Li, Chongshan Dai, Xiayun Yang, Bin Li, Xilong Xiao, Shusheng Tang

**Affiliations:** College of Veterinary Medicine, China Agricultural University, Yuanmingyuan West Road No.2, Haidian District, Beijing 100193, China; lidaowen123.fff@163.com (D.L.); daichongshan@cau.edu.cn (C.D.); fujianlw@163.com (X.Y.); lb2016@cau.edu.cn (B.L.)

**Keywords:** olaquindox, GADD45a, DNA damage, S-phase arrest, JNK/p38 pathways

## Abstract

Olaquindox, a quinoxaline 1,4-dioxide derivative, is widely used as a feed additive in many countries. The potential genotoxicity of olaquindox, hence, is of concern. However, the proper mechanism of toxicity was unclear. The aim of the present study was to investigate the effect of growth arrest and DNA damage 45 alpha (GADD45a) on olaquindox-induced DNA damage and cell cycle arrest in HepG2 cells. The results showed that olaquindox could induce reactive oxygen species (ROS)-mediated DNA damage and S-phase arrest, where increases of GADD45a, cyclin A, Cdk 2, p21 and p53 protein expression, decrease of cyclin D1 and the activation of phosphorylation-c-Jun N-terminal kinases (p-JNK), phosphorylation-p38 (p-p38) and phosphorylation-extracellular signal-regulated kinases (p-ERK) were involved. However, GADD45a knockdown cells treated with olaquindox could significantly decrease cell viability, exacerbate DNA damage and increase S-phase arrest, associated with the marked activation of p-JNK, p-p38, but not p-ERK. Furthermore, SP600125 and SB203580 aggravated olaquindox-induced DNA damage and S-phase arrest, suppressed the expression of GADD45a. Taken together, these findings revealed that GADD45a played a protective role in olaquindox treatment and JNK/p38 pathways may partly contribute to GADD45a regulated olaquindox-induced DNA damage and S-phase arrest. Our findings increase the understanding on the molecular mechanisms of olaquindox.

## 1. Introduction

**Figure 1:**
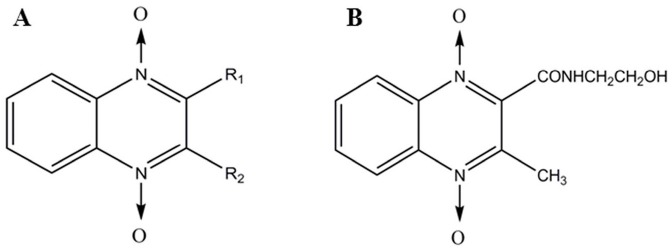
Structure of quinoxalines (**A**) and olaquindox (**B**).

Quinoxaline 1,4-dioxides (QdNOs) consisting of one or two acyclic chain moieties (Figure 1A), have many biological properties, such as antimicrobial, antitumora and anti-inflammatory actions [1,2,3]. Olaquindox (Figure 1B) is one of the QdNOs family and it has been mainly used as a growth promotant. However, in 1999, The European Commission of the European Community has forbidden the use of olaquindox as a growth promotant due to its genotoxicity and potential risk [4]. Despite a lack of evidence of toxicity and underlying mechanisms, olaquindox has still been extensively used as a feed additive in swine to improve the efficiency of feed conversion [5]. However, recently available data illustrated its potential adverse reaction such as genotoxicity and cytotoxicity [6,7], mutagenicity [8] and phototoxicity [9]. Moreover, it have been demonstrated that olaquindox had obvious cumulative toxicity [10,11]. Therefore, human and animal health may be affected because of abuse of olaquindox, such as continuous feeding, irrational dosages, or unreasonable drug withdrawal periods.

Numerous studies have shown the toxicity of olaquindox in vivo [12,13], but it is more important to evaluate the genotoxicity and cytotoxicity of olaquindox. Previous studies have shown that olaquindox could induce marked cytoxicity and genotoxicity in African green monkey cell lines (Vero cells) and HepG2 cells [7,14]. Very recently, Yang et al. [15] study demonstrated that olaquindox exerted genotoxic effects and induced DNA strand breaks in human embryonic kidney cell line 293 (HEK293) cells, and lysosomal-mitochondrial pathway mediated ROS production and p53 activation played an important role. Oxidative damage was caused by excess ROS, which has been proposed as a possible mode of action for DNA injury [16]. Oxidative stress plays an important role in QdNOs induced DNA damage in the tissues of rat or mouse [17]. In our previous study, we found that S-phase mediated cell cycle arrest participated in olaquindox-induced DNA injury [18].

A new discovery has been found whereby GADD45a influenced furazolidone-induced S-phase cell cycle arrest in HepG2 cells via cyclin D1, cyclin D3, and CDK6 [19]. GADD45a, a member of GADD45 family, have been implicated in the regulation of many cellular functions, including DNA repair, cell cycle checkpoint, signaling transduction and maintenance of genomic stability [20]. The role of GADD45a in the DNA repair machinery is still unclear. It has been reported that GADD45a-deficient mice show increased sensitivity to radiation, genomic instability and chromosome abnormalities [21]. MEFs lacking GADD45a genes exhibited decreased colony-forming ability after UV radiation and cisplatin exposure compared to wild-type MEFs, indicating their sensitivity to DNA damage [22]. Some findings suggested that the mammalian genome might be protected by a multiplicity of G2-M checkpoints so that GADD45a might play role in the control of cell cycle arrest in response to specific types of DNA damage such as UV and methyl methanesulfonate (MMS) [23]. Moreover, after UV irradiation, a pronounced S-phase accumulation of GADD45a deficient cells was observed [22]. Cyclin A and their relative protein are responsible for regulating the cell cycle transition, some relevant signaling pathways activated when the cell cycle arrest in S-phase, which regulates cell death and inhibition of cell proliferation [19,24]. It suggested that apoptosis in HepG2 cells might be suppressed through p38 MAPK and ROS-phosphorylation of JNK pathways in response to olaquindox treatment [25]. Moreover, it was well established that excessive ROS generation lead to the activation of mitogen-activated protein kinase (MAPK) in human pancreatic cancer cells [26] and human cervical cancer cells [27]. Furthermore, it has been demonstrated that GADD45a induction by nickel negatively regulates JNKs/p38 activation [28].

The HepG2 cell line is suitable for studying in vitro xenobiotic metabolism and potential hepatotoxicity due to its many specialized functions indicative of normal human hepatocytes [29]. HepG2 cells have been widely used as a tool for studying genotoxicity, oxidative stress, mitochondrial dysfunction and apoptosis. In present study, we aimed to provide a mechanistic understanding of how GADD45a regulates olaquindox-induced DNA damage and S-phase arrest in HepG2 cells. We investigated the role of GADD45a and JNK/p38 pathways in olaquindox-induced DNA damage and cell cycle arrest. Our findings shed insight into the molecular mechanisms of olaquindox. In the present study, 800 μg/mL of olaquindox was selected in the present study based on the IC_50_ of olaquindox in HepG2 cells. We established a model of toxicology in vitro and provide fundamental data for a subsequent toxicity study of olaquindox in vivo.

## 2. Results

### 2.1. GADD45a Knockdown Cell Line Identification

**Figure 2:**
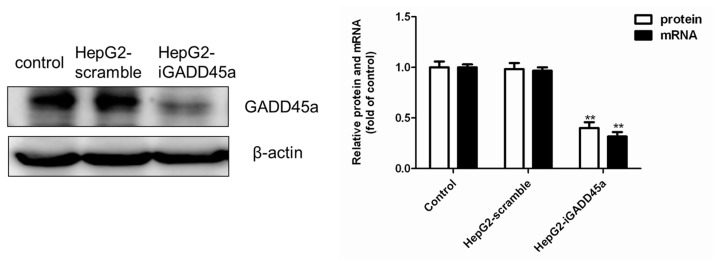
The protein and mRNA level of GADD45a was detected by western blot and qRT-PCR. β-actin mRNA amplification was used as a control for qRT-PCR. The data analysis results were exhibited in the right panels. All results were presented as mean ± SD, from three independent experiments. ** *p* < 0.01, compared with control.

As shown in Figure 2, GADD45a expression was determined by western blot and qRT-PCR. There was no significant difference between the vehicle control group and normal control group both in protein and mRNA expression. Compared with the control group, the protein and mRNA expression of GADD45a in HepG2-iGADD45a group was significantly reduced, which indicated the cell line was successfully established. GADD45a knockdown cell line successfully reduced protein and mRNA to 31% and 26% of its normal levels (Figure 2).

### 2.2. Effects of Olaquindox-Induced Cytotoxicity in HepG2 and HepG2-iGADD45a Cells

**Figure 3:**
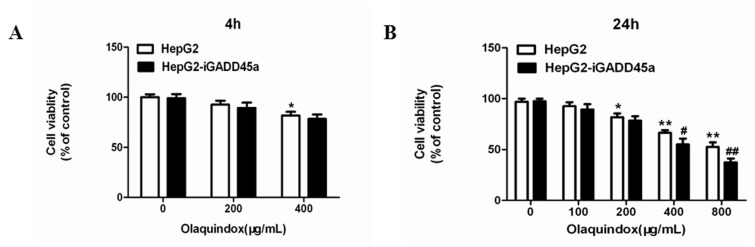
Effects of olaquindox-induced cytotoxicity determined by MTT. (**A**) Olaquindox exposed to HepG2 and HepG2-iGADD45a cells on the cell viability for 4 h; (**B**) Olaquindox exposed to HepG2 and HepG2-iGADD45a cells on the cell viability for 24 h. All results were presented as mean ± SD, from three independent experiments. (* *p* < 0.05, ** *p* < 0.01, compared with the control group; # *p* < 0.05, ## *p* < 0.01, compared to HepG2 groups).

The cytotoxicity of olaquindox exposed to HepG2 and HepG2-iGADD45a cells for 4 and 24 h was examined. At 4 h, the cell viabilities of HepG2 cells decreased to 90% and 83% in the olaquindox 200 and 400 µg/mL groups (Figure 3A). However, there was no significant difference between HepG2 and HepG2-iGADD45a cells. Furthermore, the viabilities of the cells treated with olaquindox for 24 h were more than 80% in the 100 and 200 µg/mL groups (Figure 3B).

### 2.3. Effects of GADD45a on Olaquindox-Induced DNA Damage in HepG2 Cells

**Figure 4:**
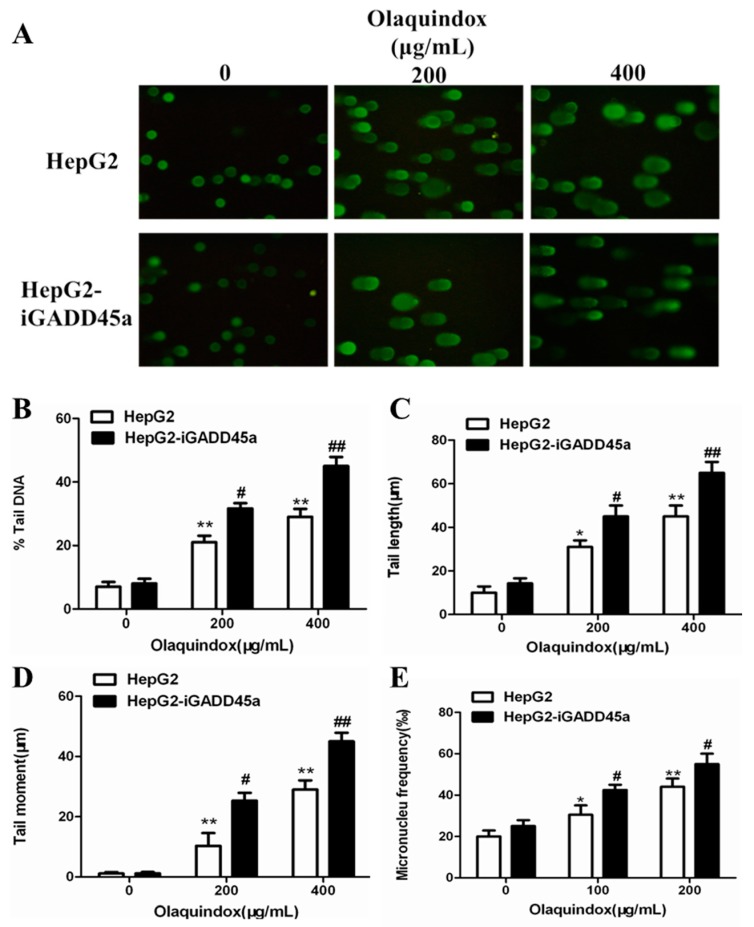
Effects of GADD45a on olaquindox-induced DNA damage in HepG2 cells. DNA strand break was measured by the comet assay. (**A**) HepG2 and HepG2-iGADD45a cells were treated with olaquindox (0, 200 and 400 µg/mL, respectively) for 4 h. Cells were observed under a Leica inverted fluorescence microscope (400×); (**B**) % tail DNA; (**C**) tail length; (**D**) tail moment; (**E**) HepG2 and HepG2-iGADD45a cells were treated with olaquindox (0, 100 and 200 µg/mL, respectively) for 24 h. 1000 binucleated cells were recorded from each experiment. All results were presented as mean ± SD, from three independent experiments. (* *p* < 0.05, ** *p* < 0.01, compared with the control group; # *p* < 0.05, ## *p* < 0.01, compared to the HepG2 groups).

Only cultures with a cell viability of more than 80% were used for comet assay analysis. Cell viability was examined using trypan blue staining at first. In all the groups, cell viabilities were more than 80%. The results obtained from the comet assay showed that olaquindox could significantly induce DNA strand breaks in HepG2 cells, as shown in Figure 4A. As for the comet result, there were no significant differences between HepG2 and HepG2-iGADD45a in 0 μg/mL olaquindox groups. Compared with the control, at the olaquindox 200 and 400 µg/mL, the percentage (%) tail DNA increased to 18.9% and 31.5%, tail DNA were detected significant increased when HepG2-iGADD45a cell were treated with olaquindox at 200 µg/mL (increased to 27.6%) and 400 µg/mL (increased to 53.9%), respectively (Figure 4B); the tail length increased to 34.3 and 54.2 µm, which were significantly increased in HepG2-iGADD45a group (increased to 43.1 and 68.6 µm) (Figure 4C); the comet tail moment values increased to 13.2 µm and 24.3 µm, which were increased in the treatment of HepG2-iGADD45a group (increased to 21.1 and 47.4 µm), respectively (Figure 4D). To further clarify that olaquindox-induced DNA damage, micronucleus assay was performed. Compared with the control, HepG2 cells treated with 100 and 200 µg/mL olaquindox for 24 h, the number of micronucleus significantly increased to 35.8‰ and 48.2‰, whereas HepG2-iGADD45a cells treated with olaquindox the number of micronucleus increased to 46.7‰ and 58.6‰ (Figure 4E).

### 2.4. The Role of ROS in Olaquindox-Induced DNA Damage

**Figure 5:**
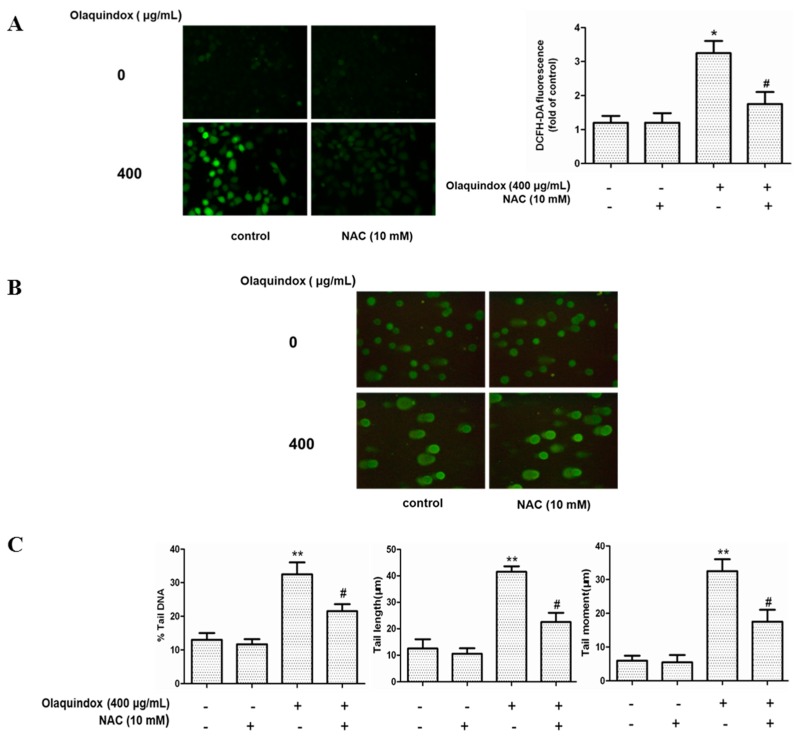
The role of ROS in olaquindox-induced DNA damage. Cells were pre-treated with NAC (10 mM) for 2 h and then co-treated with olaquindox for 24 h and then incubated with 10 μM DCFH-DA for 30 min at 37 °C. (**A**) The fluorescence intensity was visualized with a fluorescent microscope, and the images (400×) presented are representative of the fluorescence levels observed three times. The analysis of fluorescent intensity used Image Pro Plus 5.0 software; (**B**) DNA strand break was measured by the comet assay and cells were observed under a Leica inverted fluorescence microscope (400×); (**C**) The data analysis of % tail DNA, tail length and tail moment. All results were presented as mean ± SD, from three independent experiments. (* *p* < 0.05, ** *p* < 0.01, compared with the control group; # *p* < 0.05, compared to the olaquindox alone groups).

Intracellular ROS was measured by DCFH-DA fluorescence dye in the olaquindox-treated HepG2 cells. As shown in Figure 5A, compared with the control group, 400 µg/mL olaquindox treatment significantly increased the intracellular ROS to approximately 3.5-fold. Compared to the olaquindox alone group, NAC treatment abrogated olaquindox-induced ROS generation (Figure 5A). In addition, NAC also blocked olaquindox-induced DNA damage (Figure 5B,C).

### 2.5. Effects of GADD45a on the Olaquindox-Induced Cell Cycle Arrest in HepG2 Cells

**Figure 6:**
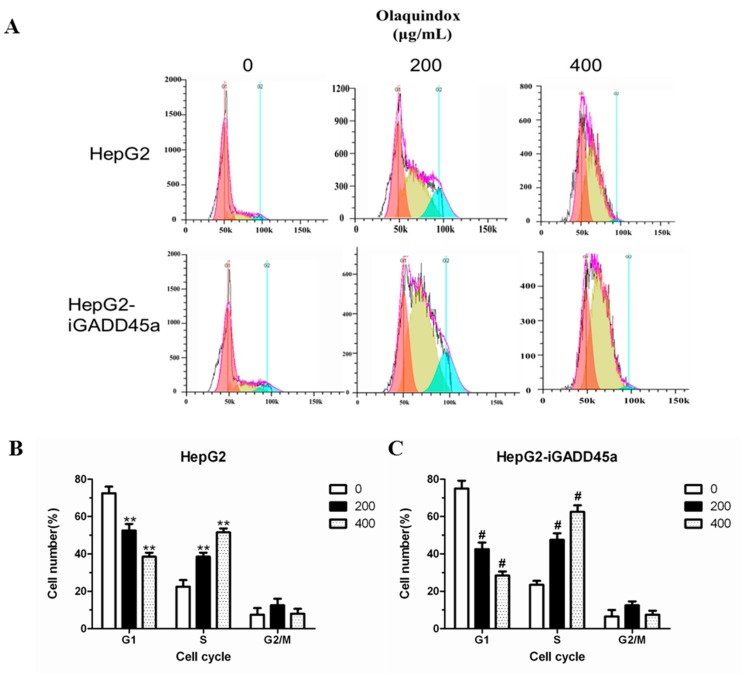
Effects of GADD45a on the olaquindox induced cell cycle arrest. in HepG2 cells (**A**) HepG2 and HepG2-iGADD45a cells were treated with olaquindox (200 and 400 µg/mL, respectively) for 24 h; (**B**) Effect of olaquindox on cell cycle distribution of HepG2 cells; (**C**) Effect of olaquindox on cell cycle distribution of HepG2-iGADD45a cells. Samples were measured by a flow cytometer. A minimum of 20,000 cells were recorded from each experiment. All results were presented as mean ± SD and three independent experiments were carried out. (** *p* < 0.01, compared with the control; # *p* < 0.05, compared to HepG2 groups).

To investigate the effect of olaquindox on the cell cycle distribution, the cell cycle profiles of HepG2 and HepG2-iGADD45a cells were analyzed by flow cytometry. Cells were exposed to 200 and 400 µg/mL olaquindox for 24 h. Compared with the control, at the olaquindox 200 and 400 µg/mL, the S-phase increased to 33.24% and 46.45% (Figure 6A,B). When olaquindox treated HepG2-iGADD45a cells for 24 h, proportion of cells in S-phase increased from 42.83% to 65.25% (Figure 6A,C).

### 2.6. Effects of GADD45a on the Olaquindox-Induced Cell Cycle Relative Protein Expression

**Figure 7:**
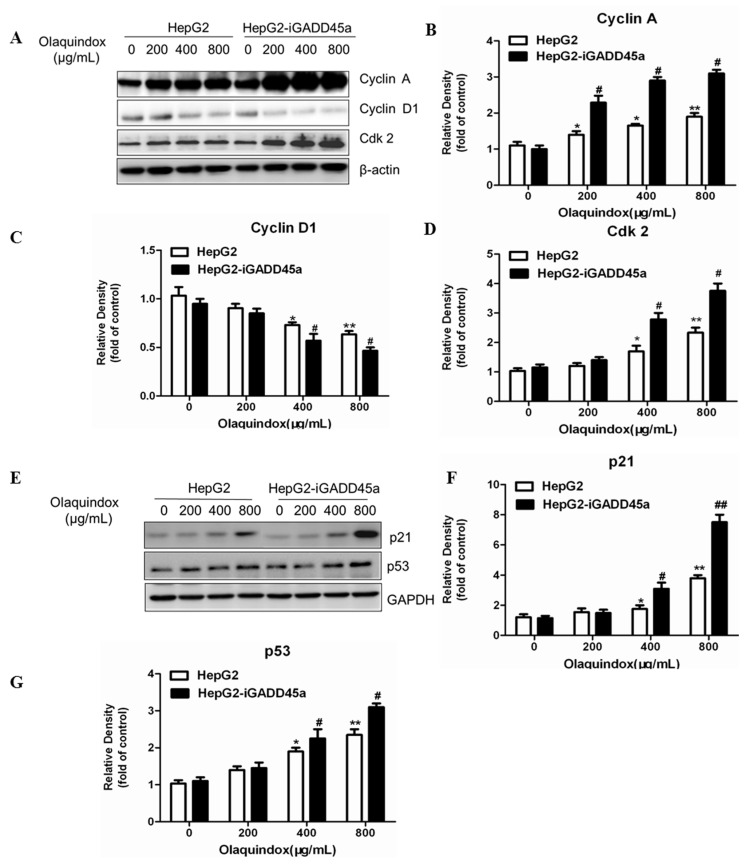
Effects of GADD45a on the olaquindox-induced cell cycle relative protein. HepG2 cells and HepG2-iGADD45a cells were exposed to 0, 200, 400 and 800 µg/mL olaquindox for 24 h. (**A**) Expression of cyclin A, cyclin D1 and Cdk2 were detected by western blotting analysis; β-actin was used for loading control. The densitometric analysis results of cyclin A, cyclin D1 and Cdk2 were shown on (**B**–**D**); (**E**) Expression of p21 and p53 were detected by western blotting; GAPDH was used for loading control. The densitometric analysis results of p21 and p53 were shown on (**F**,**G**). All results were represented as means ± SD from three independent experiments. (* *p* < 0.05, ** *p* < 0.01, compared with the control; # *p* < 0.05, ## *p* < 0.01, compared to HepG2 groups).

To further characterize olaquindox-induced S-phase arrest, we detected alteration of cyclin A, cyclin D1, Cdk2 expression. The results showed that after olaquindox treatment, cyclin A and Cdk2 expressions were increased (Figure 7A,B,D), and cyclin D1 decreased (Figure 7A,C), displaying a dose-dependent manner. Knockdown of GADD45a could significantly increase the protein expression levels of cyclin A and Cdk2, and decreased cyclin D1 (Figure 7A), which coincided with the olaquindox-induced cell cycle arrest at S-phase. Following olaquindox exposure for 24 h, compared with HepG2 group, the expression of cyclin A in HepG2-iGADD45a group markedly increased to 2.8 and 3.2-fold in the olaquindox 400 and 800 µg/mL groups; the expression of Cdk2 markedly increased to 2.9 and 3.8-fold in the olaquindox 400 and 800 µg/mL groups. Furthermore, the expression of p53 and p21 were also measured. After olaquindox treatment, the protein levels of p53 and p21 increased in a concentration-dependent manner (Figure 7E–G). However, knockdown of GADD45a in olaquindox treatment further enhanced the protein levels of p21 and p53 compared to that of HepG2 group (Figure 7E–G). Compared with those in HepG2 group, the protein levels of p21 and p53 in HepG2-iGADD45a group increased to 7.8 and 3.1-fold in the olaquindox 800 µg/mL groups, respectively.

### 2.7. Effects of GADD45a on the Olaquindox-Induced MAPKs Pathways

**Figure 8:**
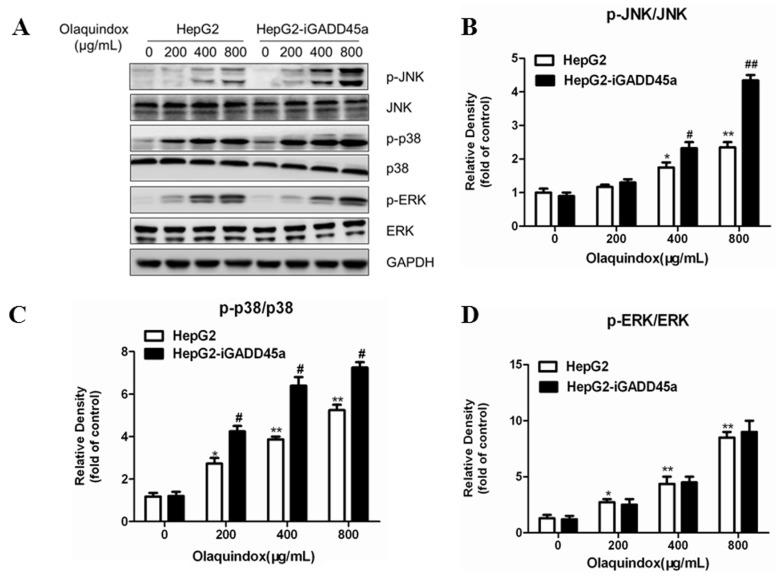
Effects of GADD45a on the olaquindox-induced MAPKs pathways (**A**) HepG2 and HepG2-iGADD45a cells were exposed to 0, 200, 400 and 800 µg/mL olaquindox for 24 h. Expression of p-JNK/JNK, p-p38/p38, p-ERK/ERK was detected by western blotting. GAPDH was used for loading control. The densitometric analysis results of p-JNK/JNK, p-p38/p38 and p-ERK/ERK were shown on (**B**–**D**). All data and results were represented as means ± SD from three or more independent experiments. (* *p* < 0.05, ** *p* < 0.01, compared with the control; # *p* < 0.05, ## *p* < 0.01, compared to HepG2 groups).

MAPKs pathways play an important role in regulating cell division, cell survival, cell apoptosis, and metabolism. Hence, we investigated the possible role of the MAPKs pathways in olaquindox treated HepG2 cells using western blotting. As shown in Figure 8A, olaquindox treatment dose-dependently resulted in the phosphorylation activation of JNK, p38 and ERK. Compared with the control, the expression of p-JNK, p-p38 and p-ERK markedly increased to 2.5, 4.6 and 7.8-fold in the olaquindox 800 µg/mL group, respectively. Knockdown of GADD45a significantly activated the phosphorylation of JNK and p38, but not ERK. Compared with the HepG2 group, the expression of p-JNK and p-p38 markedly increased to 4.3 and 6.5-fold in the olaquindox 800 µg/mL group, respectively. It indicated that JNK and p38 played an important role in GADD45a regulated olaquindox-induced DNA damage and S-phase arrest in HepG2 cells.

### 2.8. Effects of JNK/p38 Pathways on Olaquindox-Induced Cell Death

**Figure 9:**
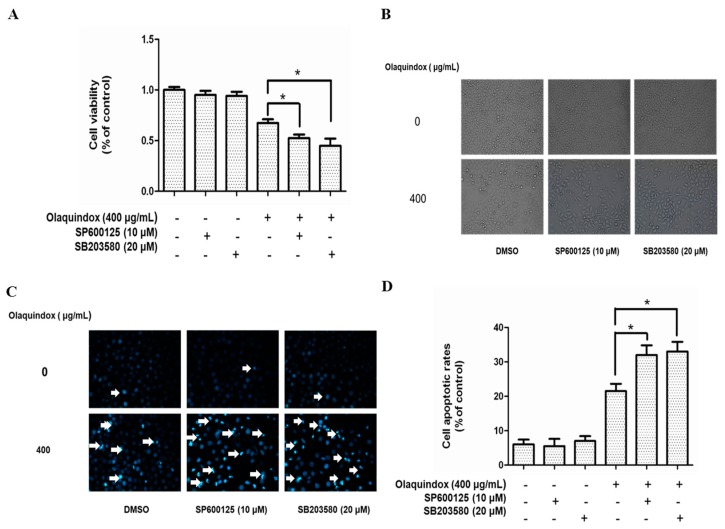
Effects of JNK/p38 pathways on olaquindox-induced cell death. HepG2 cells were pretreated with SP600125 or SB203580 for 1 h, followed to replace with or without olaquindox at the final concentration of 400 µg/mL for additional 24 h. (**A**) Cell viability was measured by MTT method; (**B**) Morphologic observation (400×); (**C**) Cell apoptosis was measured by staining with Hoechst 33342 (400×); (**D**) Quantification of apoptosis in HepG2 cells. Bright blue cells were counted as apoptotic cells from five independent microscopic fields. Results were presented as mean ± SD, from three independent experiments. (* *p* < 0.05, compared to the olaquindox alone group).

To further explore the role of JNK and p38 on olaquindox-induced growth inhibition, we examined the effects of olaquindox by using specific inhibitors on cell death. As the MTT results in Figure 9A show, treatment with SP600125 or SB203580 for 1 h, resulted in a marked decrease in cell viability. Furthermore, the morphologic observations showed that SP600125 or SB203580 treatment could induce more cell dendrite fragmentation, shrinkage, and body spindle-like (Figure 9B). As shown in Figure 9C, in HepG2 cells, compared with the control group, treatment of 400 µg/mL olaquindox alone for 24 h resulted in the appearance of a few apoptotic bodies, the shrinkage of nuclei and the condensation of chromatin. However, with pretreatment of SP600125 or SB203580, the nuclear condensation and fragmentation induced by olaquindox was significantly aggravated. Compared with the control group, olaquindox exposure effectively increased the cell apoptotic rates to 22.6%, while the pretreatment with SP600125 and SB203580 for 1 h increased the apoptosis rates to 35.5% and 36.4%, respectively (Figure 9D).

### 2.9. JNK/p38 Pathways Played Protective Role in Olaquindox-Induced DNA Damage

**Figure 10:**
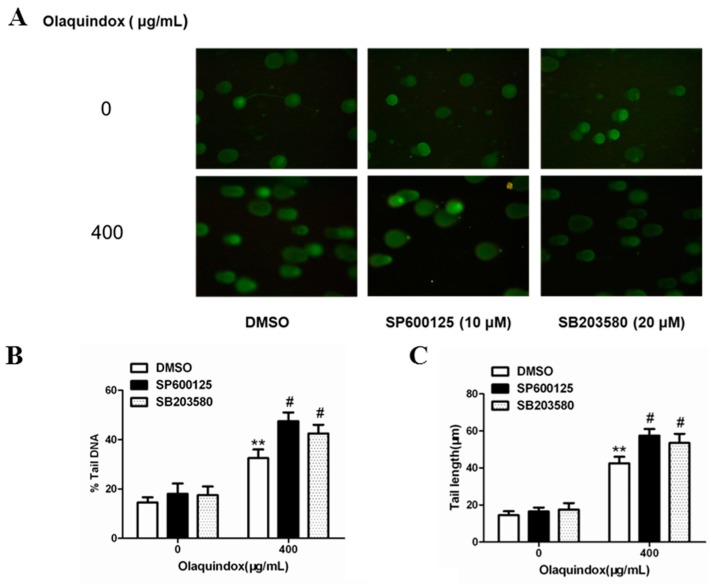

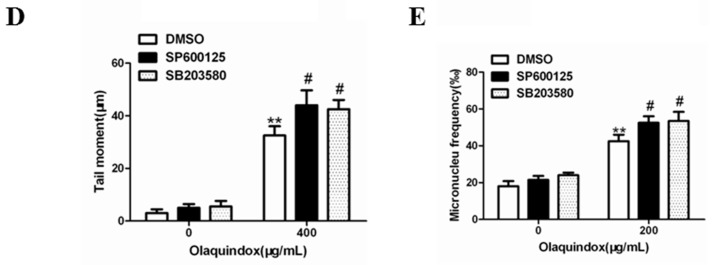
JNK/p38 pathways played protect role in olaquindox-induced DNA damage. (**A**) HepG2 cells were treated with olaquindox (400 µg/mL) for 4 h after preincubation with SP600125 or SB203580 for 1 h; (**B**) % tail DNA; (**C**) tail length; (**D**) tail moment; (**E**) HepG2 cells were treated with olaquindox (400 µg/mL) for 24 h after preincubation with SP600125 or SB203580 for 1 h. Cells were observed under a Leica inverted fluorescence microscope (400×). 1000 binucleated cells were recorded from each experiment and three independent experiments were carried out. All results were presented as mean ± SD. (** *p* < 0.01, compared with the control group; # *p* < 0.05, compared to olaquindox group).

Cell viability was examined using trypan blue stain at first. In all the groups, cell viabilities were more than 80%. As shown in Figure 10A, the results demonstrated that tail DNA (%), tail length and tail moment of HepG2 cells treated with olaquindox were effectively potentiated by the pretreatment with SP600125 or SB203580 (Figure 10B–D). Compared with olaquindox alone group, after pre-incubation with SP600125 or SB203580 for 1 h, the number of micronucleus of HepG2 cells treated with olaquindox for 24 h were both significant higher (Figure 10E).

### 2.10. JNK/p38 Pathways Inhibited Olaquindox-Induced S-Phase Arrest

**Figure 11:**
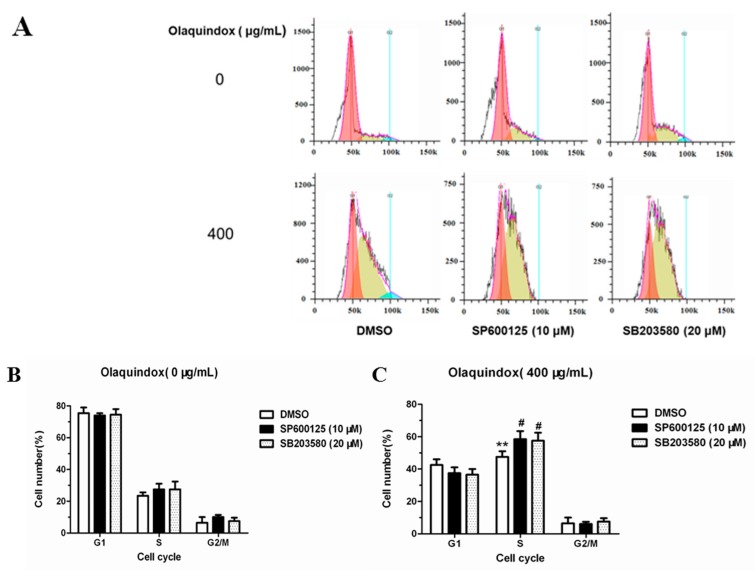
JNK/p38 pathways inhibited olaquindox-induced S-phase arrest. (**A**) HepG2 cells were treated with 400 µg/mL of olaquindox for 24 h after preincubation with SP600125 or SB203580 for 1 h; (**B**) Effect of HepG2 cells after preincubation with SP600125 or SB203580 on cell cycle distribution; (**C**) Effect of HepG2 cells treated with 400 µg/mL of olaquindox for 24 h after preincubation with SP600125 or SB203580 for 1 h on cell cycle distribution. Samples were measured by a flow cytometer. A minimum of 20,000 cells were recorded from each experiment. All results were presented as mean ± SD and three independent experiments were carried out. (** *p* < 0.01, compared with the control; # *p* < 0.05, compared to HepG2 groups).

As shown in Figure 11, S-phase arrest was more obvious after pre-incubation with SP600125 or SB203580 for 1 h in HepG2 cells treated with olaquindox for 24 h. Compared with the control group, olaquindox exposure effectively increased the S-phase to 42.1%, while the pretreatment with SP600125 and SB203580 for 1 h increased the S-phase to 57.5% and 54.4%, respectively.

### 2.11. The Relationship between GADD45a and JNK/p38 Pathways in Olaquindox Response in HepG2 Cells

**Figure 12:**
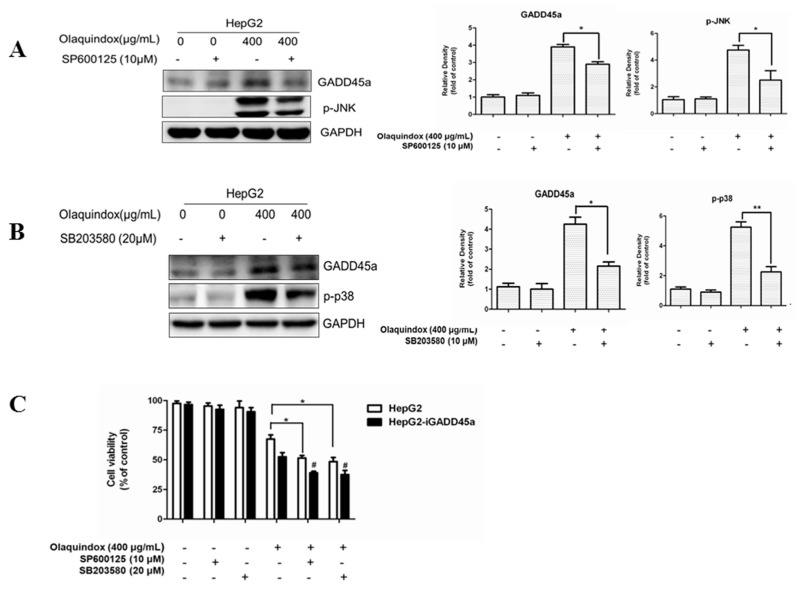
The relationship between GADD45a and JNK/p38 pathways in olaquindox response in the HepG2 cells. (**A**,**B**) Effects of SP600125 and SB203580 on the protein level of GADD45a in HepG2 cells. Cells were pre-treated with these inhibitors for 1 h before olaquindox treatment; (**C**) Effects of SP600125 and SB203580 on the cell viability both in HepG2 and HepG2-iGADD45a cells. All data and results were represented as means ± SD from three or more independent experiments. (* *p* < 0.05, ** *p* < 0.01, compared with the control; # *p* < 0.05, compared to HepG2 groups).

To further explore the role of GADD45a and JNK/p38 in olaquindox treatment, we examined the effects by using specific JNK and p38 inhibitors on the protein expression of GADD45a and cell viability. As the results shown in Figure 12, treated with olaquindox (400 µg/mL) for 24 h after pre-incubation with SP600125 or SB203580 for 1 h, apparently diminished protein expression level of GADD45a (Figure 12A,B), and resulted in a marked decrease in cell viability (Figure 12C). Furthermore, treatment with SP600125 or SB203580 in HepG2-iGADD45a cells obviously decreased cell viability compared to that of HepG2 cells induced by olaquindox (Figure 12C).

## 3. Discussion

Olaquindox is an important member of the quinoxaline family which is used as a feed additive [30]. Olaquindox has been widely used as a growth-promoting feed additive in the pig industry in China. The toxicity of olaquindox to animals and humans and the phenomenon of olaquindox abuse have been of wide concern, as the potentially toxic residues of olaquindox in edible animal-derived products could affect human health. Data from the current study verified very clearly that at a relatively low concentration, 6.6 μg/mL olaquindox expressed dramatic mutagenesis effects by having a 12-fold up-regulation in mutation frequency [8]. In our previous study, we have demonstrated that GADD45a regulated the mitochondrial apoptosis pathway in HepG2 cells treated with olaquindox [6]. In order to further research the role of GADD45a, we investigated GADD45a and JNK/p38 on olaquindox induced genotoxicity and cytotoxicity and the potential mechanism in HepG2 cells.

Our results showed that olaquindox treatment significantly attenuated cell viability both in HepG2 cells and HepG2-iGADD45a cells (Figure 3). Notably, knockdown GADD45a can significantly reduce the cell viability (Figure 3B), indicating that GADD45a may protect HepG2 cells from olaquindox-induced cytotoxicity. It has been shown that blocking GADD45a expression by constitutive antisense expression sensitized cells to be killed by UV or by cis-platinum (II) diamine-dichloride (CDDP, or cisplatin), a cancer chemotherapy drug which produces DNA cross-links [23]. The cytokinesis blocked micronucleus assay is a well-known comprehensive system that provides data from the DNA damage measures of cytostaticity and cytotoxicity while the comet assay measures DNA damage by assessing strand breaks in single cells. In our previous study, olaquindox could cause DNA double-strand breaks and micronucleus formation in HepG2 and Vero cells [7,18]. In the present study, the results showed the same findings in HepG2 cells (Figure 4). Consequently, we found that knockdown of GADD45a can aggravate olaquindox-induced DNA strand breaks (Figure 4A) and increases of micronucleus (Figure 4E), which is consistent with the previous results showing that MEFs lacking GADD45a genes exhibited sensitivity to DNA damage [31]. These results revealed that GADD45a could indeed protect cells from olaquindox-induced genotoxicity involving the inhibition of DNA damage. The possible mechanism was that GADD45a-mediated DNA repair might involve the interaction of GADD45a proteins with proliferating cell nuclear antigen (PCNA), while knockdown of GADD45a genes restrains PCNA expression in the DNA damage sites and reduces DNA-repair [32]. Cells exposed to stresses that affect cell growth or cause DNA damage, normally will undergo growth arrest until the damage is repaired; nevertheless, cells will undergo death if the damage cannot be repaired [20]. All the above results indicated that GADD45a could protect cells from olaquindox-induced DNA damage and cell death.

ROS are a normal metabolic product of cellular aerobic metabolism and excess generation of ROS contributes to DNA damage and cell death [33]. It has been testified that the main metabolic pathway of QdNOs was N→O group reduction and this could induce the generation of ROS leading to their toxicity [34]. It was inferred that olaquindox exerts genotoxic effects probably through the ROS-mediated oxidative DNA damage in HepG2 cells [18]. In the present study, we found that olaquindox exposure significantly increased intracellular ROS (Figure 5A) and caused DNA damage (Figure 5B). Meanwhile, treatment with the ROS scavenger NAC could effectively prevent the olaquindox-induced production of ROS and relieve DNA damage (Figure 5A,B). In our previous study, it has been demonstrated that GADD45a could alleviate olaquindox-induced ROS generation [6]. Taking the above-mentioned into consideration, these findings suggest that olaquindox induces ROS production directly and knockdown of GADD45a, further exacerbating ROS-mediated DNA damage.

It has been reported that after DNA damage, F-box protein FBXO31 mediates cyclin D1 degradation to induce G1 arrest [35]. In particular, the role of cell cycle checkpoints was to maintain DNA stability during the cell cycle following exposure to genotoxic agents [36], which indicated that DNA damage may by accompanied by cell cycle arrest. Induction of cell cycle arrest is one of the most effective ways to inhibit tumor growth. Some reports suggested that GADD45a played an importance role in the S-phase regulation which was further supported by research data from GADD45a-deficient mice showing arrest at the S-phase after exposure to UV radiation [37]. It has been shown that olaquindox could cause a dose-dependent progressive increase in the population of cells in S-phase and a dramatic decrease in the percentage of cells in G1 phase, but had no significant effects on the G2/M phase [38], which was consistent with the present study (Figure 6A). Furthermore, knockdown of GADD45a significantly increased in the population of cells in S-phase, compared to normal HepG2 cells (Figure 6B,C). The results indicated that GADD45a could relieve olaquindox-induced S-phase arrest. The initiation and precise regulation of cell cycle phases is choreographed by a unique and complex signal transduction system. Cyclin A and CDK2 could form a complex for the promotion of the cell cycle, regulating cell cycle progression from S-phase to G2/M [39]. This study demonstrated that olaquindox can restrict HepG2 cells in S-phase along with cyclin A and Cdk2 expression increase. As an inhibitor of cell proliferation and upstream of p53, p21 plays an important role in cell cycle arrest [40]. p53, as a cell cycle checkpoint protein, can suppress DNA synthesis, arrest cell cycle progress and inhibit cell division to maintain genetic stability [41]. The up-regulation of p21 and p53 indicated cell cycle arrest and DNA damage occurrence. Our results also showed that knockdown of GADD45a remarkably increased p21 and p53 expression (Figure 7E). These results implied that GADD45a regulated that olaquindox promoted intracellular ROS generation and activated DNA damage, and eventually caused cell cycle arrest to inhibit cells growth.

In our previous study, it has been demonstrated the levels of phosphorylation of JNK significantly suppressed after pretreatment of the antioxidants, while inhibition of the activations of JNK or p38 MAPK had no effect on ROS generation [25]. This result suggested that ROS might be the upstream mediator for the activation of JNK/p38. Several previous studies have confirmed that GADD45a was closely linked to the MAPK pathways [42,43]. The results showed that the expression of p-JNK, p-p38 and p-ERK in HepG2 cells were up-regulated after treatment with olaquindox for 24 h. Interestingly, after treatment with olaquindox in GADD45a knockdown cells, the protein levels of p-JNK and p-p38 were increased in a concentration-dependent manner, but not p-ERK, compared with HepG2 cells (Figure 8A). These data suggested that the inhibitory effect of GADD45a on JNK/p38 activation might be via up-regulation of phosphatase of MKK, rather than down-regulating upstream kinase [28]. A variety of studies have showed the multiple roles of MAPK signaling molecules as anti-apoptotic, pro-apoptotic, or non-apoptotic [26,44,45]. Previous study has been demonstrated that MAPKs inhibitors further enhance the apoptotic effect in DADS-treated HepG2 cells [46]. In present study, olaquindox induced apoptosis was significantly potentiated by the JNK inhibitor or the p38 inhibitor (Figure 9C,D). In addition, JNK inhibitor and p38 inhibitor could aggravate olaquindox induced DNA damage and S-phase arrest (Figure 10 and Figure 11). The results implied that the activation of JNK/p38 pathways played protective role in olaquindox treatment. Our results also demonstrated that there was a mutual regulation between GADD45a and JNK/p38 (Figure 8A and Figure 12A,B). These results indicated that JNK/p38 pathways partly contributed to GADD45a regulated olaquindox-induced DNA damage and S-phase arrest. In this current report, we expanded our investigation to assess that the roles of JNK and p38 may control cell death and cell cycle checkpoint via GADD45a-JNK/p38 pathway. The GADD45 family proteins are known to function as specific activators of MTK1, a MAPK kinase upstream in the p38 and JNK pathways [47]. GADD45 proteins also interact with Cdk, resulting in inhibition of kinase activity of the cdc/cyclin complex, which is a key regulator of the cell cycle [48].

**Figure 13:**
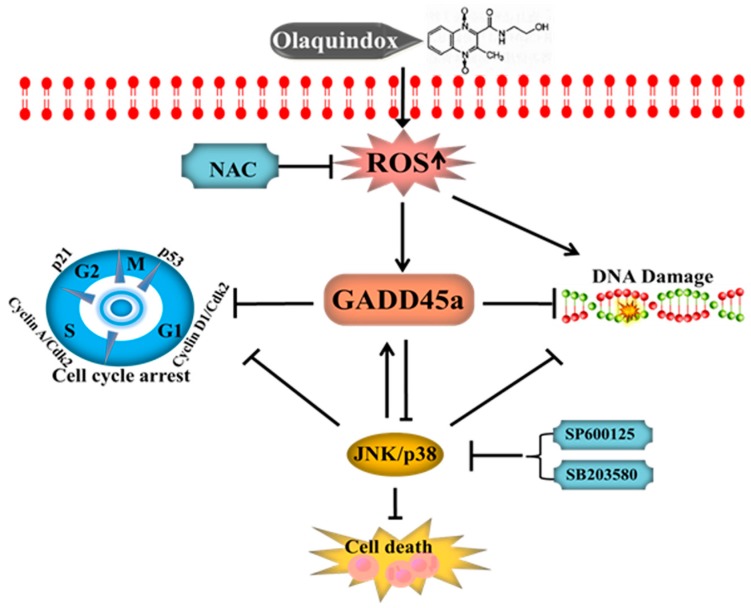
A schematic diagram of the protective effect of GADD45a on olaquindox induced DNA damage and cell cycle arrest in HepG2 cells. Olaquindox could induce the exceeding reactive oxygen species (ROS) in the metabolic process, subsequently induced the DNA damage and activated GADD45a and JNK/p38 pathways. The activation of JNK/p38 may play a protective role in olaquindox induced cell death, DNA damage and cell cycle arrest. GADD45a could effective inhibit olaquindox induced DNA damage and S-phase arrest in HepG2 cell and JNK/p38 pathways may partly contribute to GADD45a regulated olaquindox-induced DNA damage and S-phase arrest.

In summary, our findings show that olaquindox could cause obvious cytotoxicity and genotoxicity in HepG2 cell and knockdown of GADD45a could significantly aggravate olaquindox-induced cytotoxicity, DNA damage and cell cycle arrest (a schematic diagram of the proposed mechanisms is given in Figure 13). Inhibition of JNK/p38 pathways suppressed GADD45a expression and promoted olaquindox-induced apoptosis, DNA damage and S-phase arrest. JNK/p38 pathways partly contributed to GADD45a regulated olaquindox-induced DNA damage and S-phase arrest. This finding would contribute to understanding the molecular toxicity of olaquindox and other QdNOs family members and provide valuable data for rational use of this drug.

## 4. Materials and Methods

### 4.1. Materials

Olaquindox (C_12_H_13_N_3_O_4_, MW 263.25, CAS NO.23696-28-8, purity ≥98%) was supplied by the China Institute of Veterinary Drug Control (Beijing, China). Fetal bovine serum (FBS) and Dulbecco’s modified Eagle’s medium (DMEM) were obtained Gibco (Grand Island, NY, USA). RNase A and propidium iodide (PI), *N*-acetylcysteine (NAC) and 2′,7′-dichlorofluorescensindiacetate (DCFH-DA) were purchased from Beyotime (Beyotime Institute of Biotechnology, Haimen, China). 3-(4,5-Dimethyl-2-thiazolyl)-2,5-diphenyl-2*H*-tetrazolium bromide (MTT), dimethyl sulfoxide (DMSO), Triton X-100, Tween-20, sodium dodecylsulfonate (SDS), phenylmethyl sulfonyl fluoride (PMSF), JNK inhibitor (SP600125) and p38 inhibitor (SB203580) were all obtained from Sigma-Aldrich L.L.C. (St. Louis, MO, USA). All other chemicals and reagents were of reagent grade.

### 4.2. Cell Culture and Olaquindox Treatment

HepG2 cell line was obtained from the American Type Culture Collection (Manassas, VA, USA). GADD45a knockdown cell line (HepG2-iGADD45a) and negative control cell line (HepG2-scramble) were constructed in our previous study [6]. Cells were cultured in DMEM (Gibco, Grand Island, NY, USA) containing 1% penicillin and streptomycin (Beyotime Institute of Biotechnology Co., Ltd., Haimen, China), 2% l-glutamine, 10% fetal bovine serum (Gibco), at 37 °C in a humidified atmosphere of 95% air and 5% CO_2_. Olaquindox was dissolved in DMEM to make a concentration of 800 µg/mL and diluted to different concentrations with the cell culture medium, respectively, according to our previous study [18].

### 4.3. Measurement of Cell Viability

Cell viability was examined by using MTT method according to the previous study [49]. In brief, cells were (2–5 × 10^4^) seed into 96-well plates. After growth for 24 h, cells were exposed to the different concentrations of olaquindox and inhibitors for additional 4 h or 24 h. Then, the medium was removed and cells were incubated in the 100 μL fresh medium supplemented with 10 μL MTT (5 mg/mL) dissolved in culture medium for 4 h at 37 °C. Then, the media was discarded and 100 μL DMSO was added into each well to dissolve the formazan crystals at 37 °C for 30 min in the dark. The optical density was read at 570 nm in a microplate reader (Molecular Devices, Sunnyvale, CA, USA). Absorbance values presented by control correspond to 100% cell viability. At least three independent experiments were repeated.

### 4.4. Comet Assay

The comet assay was conducted according to the previous study [50]. Comet assay was performed using an Oxiselect Comet Assay^®^kit (Cell Biolabs, San Diego, CA, USA) according to the manufacturer’s instructions. Briefly, HepG2 and HepG2-iGADD45a Cells were exposure to 200 and 400 µg/mL olaquindox for 4 h and HepG2 cells were treated with 400 µg/mL of olaquindox for 4 h after preincubation with SP600125, SB203580 for 1 h. Cells were pre-treated with NAC (10 mM) for 2 h and then co-treated with olaquindox for 24 h. In all groups, the cell viability was more than 80%. Cells were collected and re-suspended in cold PBS. The cells put into the low melting agarose and subsequently loaded to the ComeSlide™. These mixes of cell-agarose were solidified at 4 °C, and immersed in a cold lysing solution (2.5 M NaCl, 100 mM Na_2_EDTA, 10% DMSO, 1% TritonX-100, pH 10) for 1 h at 4 °C. Subsequently, the slides were transferred to electrophoresis solution (1 mM Na_2_EDTA, 300 mM NaOH, pH 13) for electrophoresis at 25 V, 300 mA for 25 min at 4 °C. The slides were neutralized with 0.4 M Tris-HCl (pH 7.5). After that, the slides were washed by DI water twice and dehydrated in 70% ethanol. After staining with Vista Green DNA dye for 10 min, the image of comet was observed using a fluorescence microscopy (Leica Microsystems, Wetzlar, Germany). 100 cells per sample were obtained and recorded by Comet Assay Software Project (CASP) 1.2.2 (University of Wroclaw, Wroclaw, Poland).The tail DNA % is calculated as (tail DNA intensity/cell DNA intensity) × 100; the tail length is the length of the tail (in pixels); the tail moment length is the length from the center of the head to the center of the tail.

### 4.5. Cytokinesis Blocked Micronucleus Assay

The evaluation of micronucleus was performed using the criteria established cytokinesis blocked micronucleus assay conducted by previous study [51]. Cells were seeded at a density of 2–5 × 10^4^ into 6-well plate for 24 h. HepG2 and HepG2-iGADD45a cells were exposure to 100 and 200 µg/mL olaquindox for 24 h. HepG2 cells were treated with 400 µg/mL of olaquindox for 24 h after preincubation with SP600125 or SB203580 for 1 h. Subsequently, cells were washed twice with PBS and cultured with DMEM containing cytochalasin B (4.5 mg/mL) for 24 h. The results were expressed as the number of micronucleus in 1000 binucleated cells, and evaluated using a light microscope (Leica Microsystems).

### 4.6. Cell Cycle Assay

HepG2 and HepG2-iGADD45a cells (1 × 10^5^) were seeded into 6-well plates and exposed to olaquindox at concentrations of 0, 200, 400 µg/mL for 24 h. HepG2 cells were treated with olaquindox (400 µg/mL) for 24 h after preincubation with SP600125 or SB203580 for 1 h. Then, cells were collected after digestion with 0.05% trypsin, and fixed in 70% ice-cold ethanol at 4 °C for more than 18 h. After resuspended in PBS, cells were incubated with RNase A (100 µg/mL) for 30 min at 37 °C. Subsequently, cells were stained with 50 mg/mL propidium iodide (PI) for 30 min in the dark at room temperature. Cells were detected by FACSAria™ flow cytometry (BD Biosciences, San Jose, CA, USA). For each analysis, at least 20,000 events was collected and analyzed using FlowJo software (Tree Star, Ashland, OR, USA).

### 4.7. Western Blotting Assay

After olaquindox treatment, cells were collected and lysed in a lysis buffer (50 mM Tris-HCl, 2% SDS, 150 mM NaCl, 1 mM EDTA, 50 mM NaF, 0.5 mM Na_3_VO_4_ and 1 mM PMSF) for 15 min at 4 °C. Cell lysates were centrifuged at 12,000 rpm at 4 °C for 15 min. Cellular protein was loaded into sodium dodecyl sulfate-polyacrylamide gel (SDS-PAGE) for electrophoresis. After that, proteins were transferred to nitrocellulose membranes (Mini-Protean and Trans-Blot systems, Bio-Rad Laboratories, Hercules, CA, USA). The membranes were blocked with non-fat milk for 2 h. After being washed with tris buffered saline tween (TBST), membranes were incubated with specific primary and secondary antibodies. The membranes were detected using western luminescent detection kit (Vigorous Biotechnology, Beijing, China). Rabbit polyclonal antibodies against cyclin A (1:1000) and cyclin D1 (1:500), mouse monoclonal antibody against GADD45a (1:500) were all purchased from Santa Cruz (Santa Cruz, CA, USA). Rabbit polyclonal antibody against CDK 2 (1:1000) was acquired from ABclonal (ABclonal Biotech Co. Ltd., Cambridge, MA, USA). Mouse monoclonal antibody against p38 (1:1000) and phospho-p38 (1:1000), rabbit monoclonal antibody against JNK (1:1000), phospho-JNK (1:1000), ERK (1:1000) and phospho-ERK (1:1000) were purchased from Cell Signaling Technology (Beverly, MA, USA). p21 (1:1000), p53 (1:1000), GAPDH (1:1000) and β-actin (1:1000) were obtained from Zhongshan Golden Bridge (Beijing, China). The secondary antibodies were rabbit anti-mouse IgG (1:5000) or goat anti-rabbit IgG (1:5000) (Zhongshan Golden Bridge Co., Beijing, China).

### 4.8. Quantitative Real-Time (qRT)-PCR Analysis

Total RNA was extracted using TRlzol^®^ reagent (Life Technologies, Grand Island, NY, USA) according to the manufacturer’s instructions. qRT-PCR was carried performed using an AB7500 real-time PCR instrument (Applied Biosystems, Foster City, CA, USA). Reactions were carried out using AceQ qPCR SYBR Green Master Mix (Vazyme Biotech Co., Ltd., Nanjing, China). The PCR primers used as the following: GADD45a: forward, 5′-TGCGAGAACGACATCAACAT3-3′, reverse, 5′-GAATAAACAAAAACGGCCCT-3′; β-actin: forward, 5′-CGGGAAATCGTGCGTGAC-3′, reverse, 5′-CAGGAAGGAAGGCTGGAA GAG-3′. qRT-PCR was performed using a Chromo 4™ instrument (Bio-Rad) and the cycling conditions used were as follows: 95 °C for 10 min; 40 cycles of 95 °C for 15 s, 60 °C for 1 min, and 72 °C for 40 s. All reactions were conducted in triplicate. The fold change in gene expression was calculated using 2^−ΔΔ*C*t^ after normalizing to the expression level of β-actin.

### 4.9. Measurement of Intracellular ROS Generation

The intracellular ROS was measured with cell-permeant probe DCFH-DA. Cells were pre-treated with NAC (10 mM) for 2 h and then co-treated with olaquindox for 24 h and then incubated with 10 μM DCFH-DA for 30 min at 37 °C in the dark prior to harvest, and then washed with PBS twice. The fluorescence intensity of the cells was observed by fluorescent microscope.

### 4.10. Measurement of Apoptosis

Cell apoptosis was measured using Hoechst 33342 staining. In brief, HepG2 cells (1 × 10^5^) were cultured on 6-well culture plates and pretreated with SP600125 or SB203580 for 1 h at 37 °C. After that, the cells were washed with PBS twice and treated with olaquindox at 400 µg/mL for additional 24 h. Cells in the negative control group were treated with 0.1% DMSO for 1 h. For Hoechst 33342 staining, the treated HepG2 cells were stained with 1 µg/mL Hoechst 33342 (Vigorous Biotechnology, Beijing, China) for 20 min in the dark, then observed under a fluorescence microscope (Leica Microsystems).

### 4.11. Statistic Analysis

All data are expressed as the mean ± standard deviation (SD) at least three independent experiments. Figures were performed by Graph Pad Prism 5.0 (GraphPad Software, Inc., La Jolla, CA, USA). The data of control and treatment groups were analyzed with one way analysis of variance (ANOVA), followed by the LSD post hoc test (SPSS 17.0, Chicago, IL, USA). The level of *p* < 0.05 was considered as significant.
